# Supplementary figures and images for: Glycosylation Analysis of Feline Small Intestine Following Toxoplasma gondii Infection
Source: Animals (Basel). 2022 Oct 20;12(20):2858. doi: 10.3390/ani12202858 (PMC9597833; doi:10.3390/ani12202858)

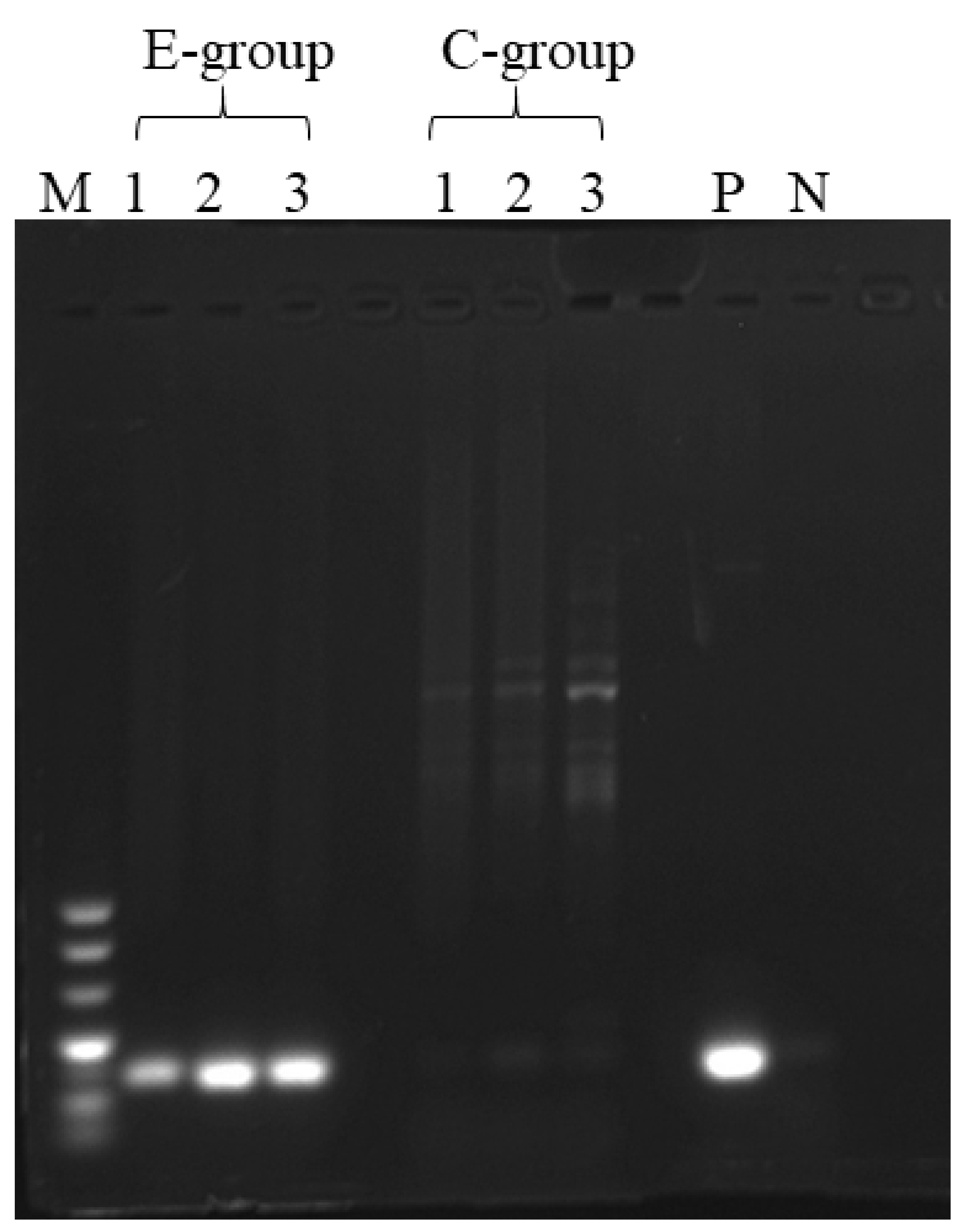

Supplement: Supplementary file 1 [file animals-12-02858-s001.zip › Supplementary Files/Supplementary figures 9.3/Supplementary Figure S1.tif]

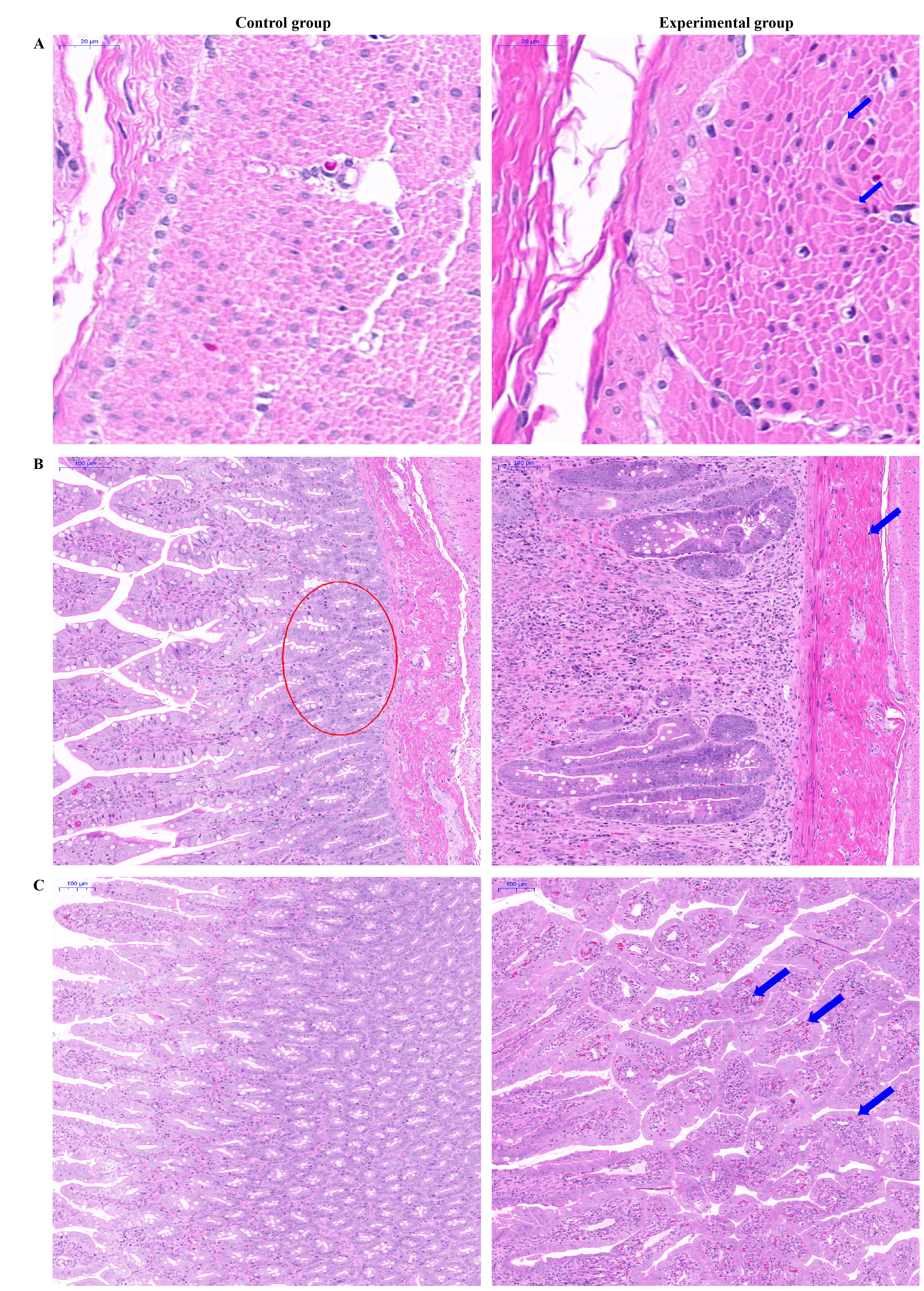

Supplement: Supplementary file 1 [file animals-12-02858-s001.zip › Supplementary Files/Supplementary figures 9.3/Supplementary Figure S2.jpg]

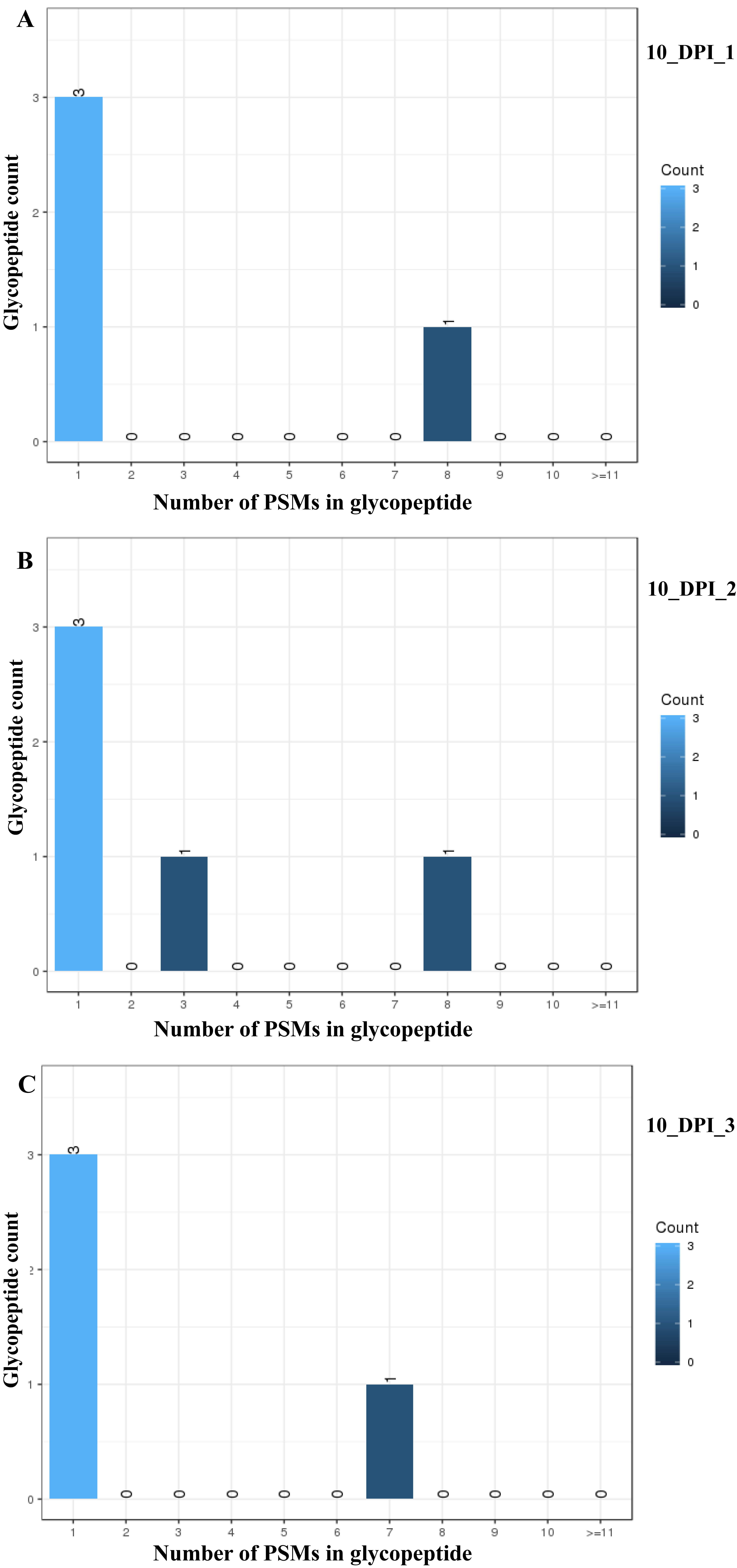

Supplement: Supplementary file 1 [file animals-12-02858-s001.zip › Supplementary Files/Supplementary figures 9.3/Supplementary Figure S3.tif]

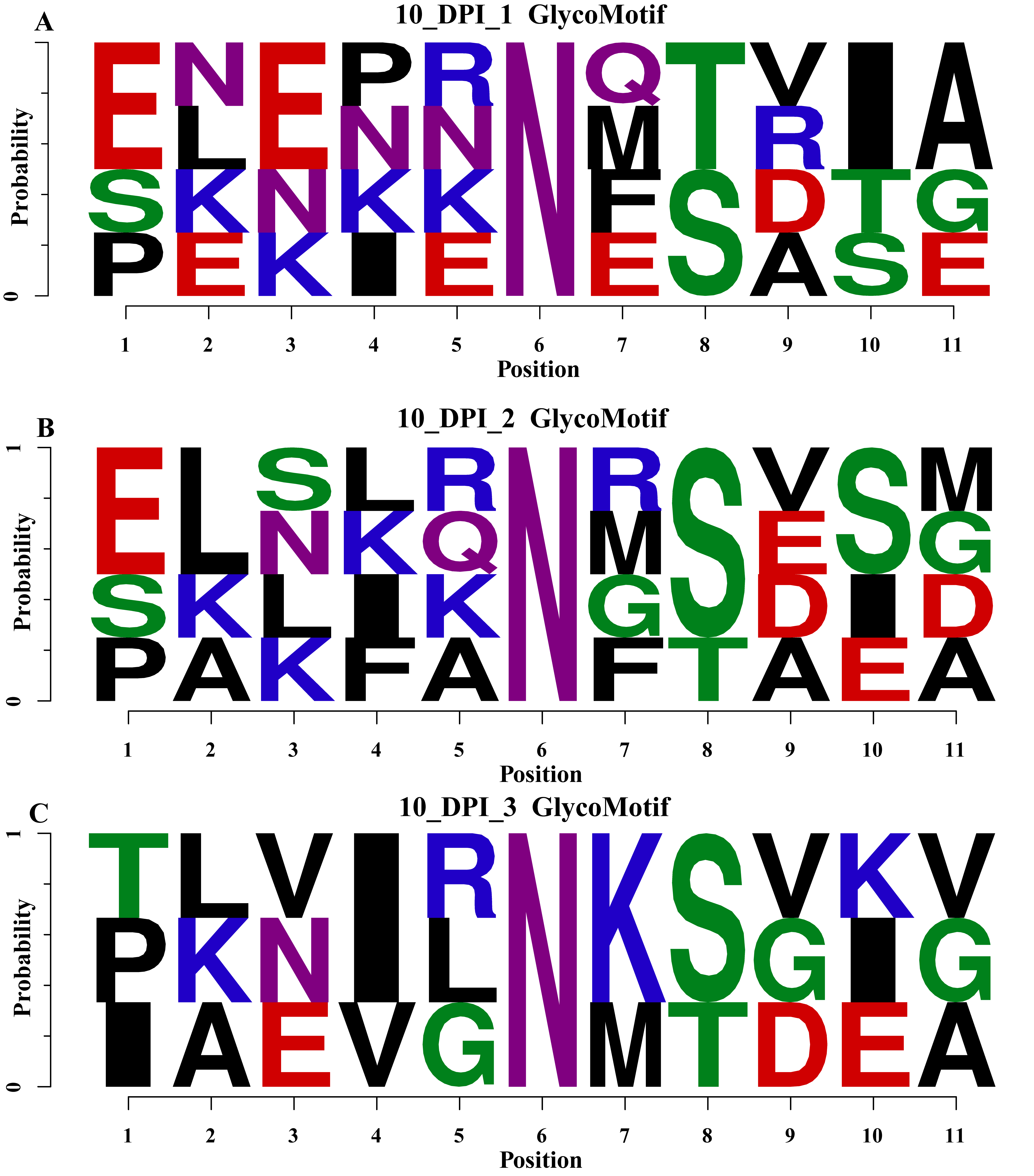

Supplement: Supplementary file 1 [file animals-12-02858-s001.zip › Supplementary Files/Supplementary figures 9.3/Supplementary Figure S4.tif]
